# Treatment of Tricuspid Regurgitation With the FORMA Repair System

**DOI:** 10.3389/fcvm.2018.00140

**Published:** 2018-10-15

**Authors:** Gidon Y. Perlman, Danny Dvir

**Affiliations:** ^1^Hadassah Medical Center, Jerusalem, Israel; ^2^Cardiology Department, University of Washington, Seattle, WA, United States

**Keywords:** tricuspid regurgitation (TR), transcatheter, effective regurgitant orifice, FORMA, right ventricle (RV)

## Abstract

**Background:** Tricuspid regurgitation (TR) is common and undertreated as the risk of surgery is high in this patient population. Transcatheter devices offer treatment with a lower procedural risk. The FORMA Tricuspid Valve Therapy system (Edwards Lifesciences) will be reviewed here.

**Device Description:** The system combines a spacer placed in the regurgitant orifice and a rail, over which the spacer is delivered, that is anchored to the endocardial surface of the RV. The spacer provides a surface for leaflet coaptation.

**Outcomes:** Eighteen compassionate care patients and 29 patients included in the US EFS trial are reviewed. Patients were elderly (76 years) and high risk (Euroscore 2 was 9.0 and 8.1%, respectively). There were 2 procedural failures in both groups. Mortality at 30 days was 0% in the compassionate group and 7% in the EFS trial. TR was reduced in both groups; 2D/3D EROA 2.1 ± 1.8 to 1.1 ± 0.9 cm^2^ in the EFS trial and vena contracta width 12.1 ± 3.3 to 7.1 ± 2.2 mm. Symptomatic improvement was seen in both groups; the proportion of patients in NYHA class III/IV decreased from 84 to 28% at 30 days in the EFS group, and from 94 to 21% at 1 year, in the compassionate group.

**Conclusions:** Reduction of TR with FORMA system is feasible and sustained. Despite residual TR post-procedure, the significant relative reduction in TR severity contributes to substantial clinical improvements in patients with a FORMA device in place.

## Introduction

Severe tricuspid regurgitation (TR) is a common and under-treated valvular pathology associated with increased morbidity and mortality (1–4). Recent data has shown that despite advances in surgical care over the years, surgery for isolated TR is rarely performed and operative mortality is still discouraging (5, 6).

Transcatheter therapies for TR have the potential advantage over surgery of reduced procedural risks, mainly due to reduced bleeding (from venous access sites) and reduced thoracic tissue damage, enabling easier recovery. Current transcatheter devices treat TR by improving leaflet coaptation or by modulating the annular geometry (7–12). The tricuspid valve apparatus is adjacent to both the right coronary artery (RCA) and the AV node; furthermore, the right atrium (RA) and right ventricle (RV) are thin walled and prone to injury from transcatheter devices. Performing percutaneous procedures for TR requires careful maneuvering around these structures to avoid injury, which has been reported after surgery (13) and with transcatheter devices (10, 14).

Patients with severe TR tend to have important co-morbidities. TR is associated with renal dysfunction that is often exacerbated by diuretics and hepatic congestion caused by TR can lead to liver dysfunction. Atrial fibrillation is strongly associated with TR and patients often require anticoagulation for prevention of thromboembolic events. These conditions combine to put patients with severe TR at an increased risk of bleeding. Furthermore, chronic edema of the lower extremities which is the hallmark of right sided failure and contribute to significant impairment of ambulation. Taken together, the comorbid conditions of patients with severe TR can greatly impact the recovery and outcomes of patients after transcatheter TR repair.

The FORMA Tricuspid Valve Therapy System (Edwards Lifesciences, Irvine, CA) is a transcatheter device for treatment of patients with severe secondary TR. This review will present accumulating data on short and mid-term results of patients treated with this device.

### Device description

**Figure 1:**
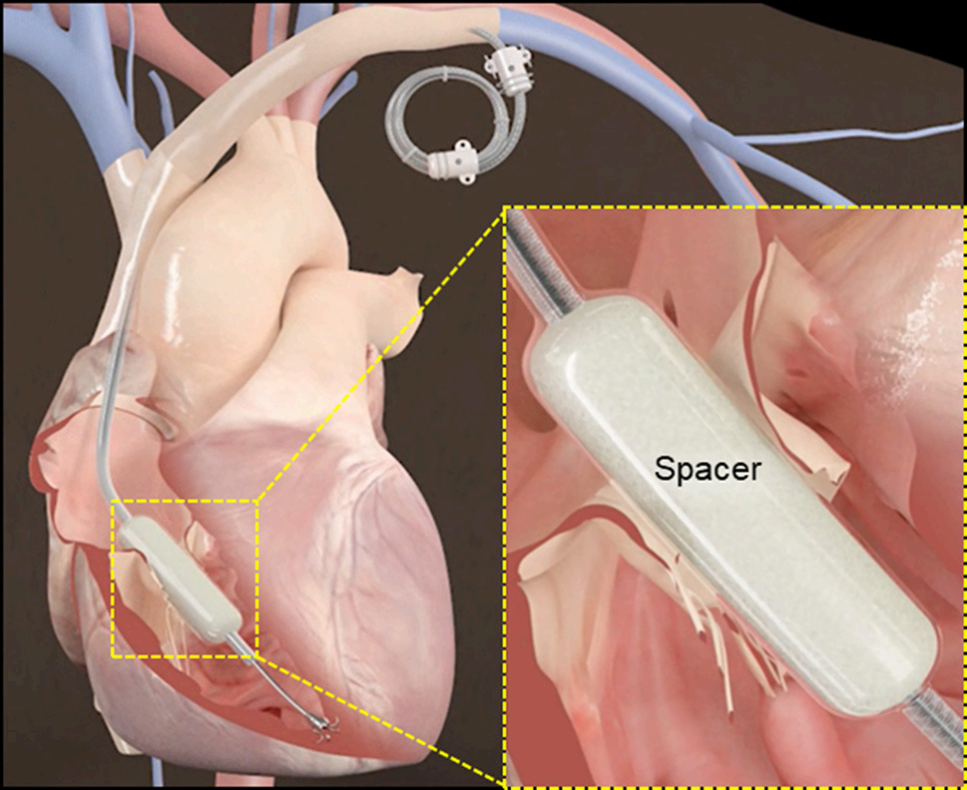
Schematic representation of the FORMA system.

The Edwards FORMA™ system combines a spacer unit placed in the regurgitant orifice and a rail, over which the spacer is delivered, that is anchored to the endocardial surface of the RV (Figure 1). The spacer is a foam-filled polymer balloon that is round and tubular shaped, it is 42 mm long and has diameters of 12, 15, and 18 mm. When the spacer is positioned across the tricuspid annulus the native leaflets have a new surface for coaptation, thereby improving TR. Insertion of the spacer across the valve is done over a rail; the rail extends from the left subclavian vein to the RV apex. A nitinol anchor with 6 curved prongs is designed to grasp the RV myocardium without exiting into the pericardial space.

A 20F or 24F sheath is required for implantation of the FORMA system, through the sheath a dedicated delivery system is used to position the anchor and rail in the correct position. The delivery system can be flexed to navigate the anchor through valve and RV trabeculations. Close to the tip of the delivery system is a large balloon which is inflated prior to crossing the valve, to avoid entanglement in the valve chordae. The rail and spacer are fully retrievable using a specially designed retrieval system.

### Implantation process

The FORMA system is implanted under general anesthesia with transesophageal echocardiographic (TEE) monitoring, the details of the procedural steps have been previously reported (7, 14). Kodali recently reported the procedural outcomes of the US early feasibility study (EFS), procedural time in this group of patients was 110 min (15).

**Figure 2:**
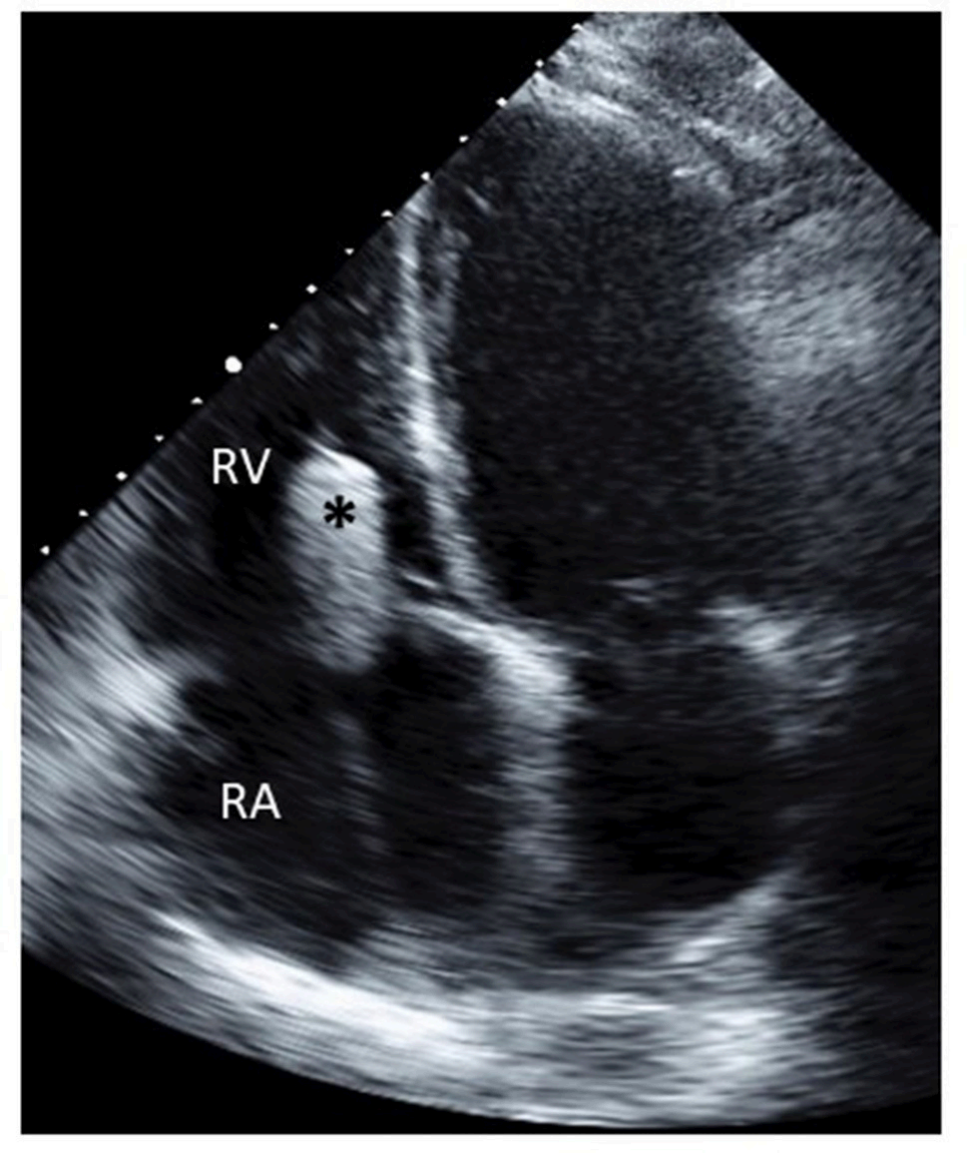
Transthoracic echocardiography showing the FORMA spacer (*) positioned in the tricuspid valve.

The procedure is divided to 4 main steps:

1. Venous access—Access to the left subclavian vein can be achieved by surgical cut-down or percutaneously with pre-closure with ProGlide (Abbott vascular) sutures. Patients with pre-existing pacemakers/defibrillators can be treated even if the generator is in the left delto-pectoral groove. Most patients with severe TR have congested veins that can accommodate the 20F or 24F sheath. Indwelling pacemaker leads can occasionally occlude the left venous system, can impede implantation of the FORMA system. The sheath is advanced to the left innominate vein/superior vena cava junction to support the delivery catheter.
2. Anchoring—The anchor target is on the lateral wall of the RV close to the interventricular septum. The anchor is positioned perpendicular to the annular plane to ensure optimal coaptation of the tricuspid valve leaflets. Manipulation of the delivery catheter in the RV is performed under fluoroscopic and TEE guidance to direct the catheter to the anchor site. TEE imaging of the anchor site is important to verify correct positioning and engagement of the anchor prongs in myocardial tissue.
3. Spacer positioning—After the rail is anchored to the RV the spacer slides down it to straddle the tricuspid annulus. The spacer self-centers in the regurgitant orifice, coaptation of the leaflets can be improved by minor adjustments of the spacer location and along the rail. When echocardiography and hemodynamic monitoring confirm adequate reduction of TR the position of the spacer is locked on the rail (Figure 2).
4. Rail fixation and access closure—The length of the rail is trimmed, and its proximal end is sutured to the subcutaneous tissue of the delto-pectoral groove to fixate the rail and the spacer. Venous closure is achieved either surgically or percutaneously.

### Patient population

Data on patient characteristics has been reported for 18 patients treated under compassionate care conditions (14) and 29 patients included in the US EFS trial (15). These patient populations can be characterized as high surgical risk. The Society of Thoracic surgeons (STS) score (calculated for mitral valve replacement) in the EFS trial was 9.1 ± 6.8% and the Euroscore 2 score was 8.1 ± 5.3%. The Euroscore 2 score of the compassionate care patients was 9 ± 5.7%. Patients in both cohorts were elderly (mean age 76) and predominantly women (66 and 72%). Multiple co-morbidities were common and prior left-sided surgery was present in approximately half (50 and 48.3%). Atrial fibrillation was present in 89% of the compassionate and 83% of the EFS trial patients. Pacemakers were present in 3/18 (17%) of the compassionate care and 7/29 (24%) of the EFS trial patients. Baseline characteristics are shown in Table 1.

**Table 1:** Baseline characteristics.

**Characteristic**	**Compassionate care (*n* = 18)**	**EFS trial (*n* = 29)**
Age, years	76 ± 9.7	75.9 ± 8.2
Female sex–No. (%)	13 (72)	19 (66)
Serum creatinine-mg/dl	1.5 ± 0.8	1.3 ± 0.4
NYHA functional class III/IV–No. (%)	17 (94)	25 (86)
EuroSCORE II	9 ± 5.7	8.1 ± 5.3
STS (for mitral valve replacement)	NA	9.1 ± 6.8
6 min walk test-meters^*^	256 ± 103	183 ± 96
Kansas City cardiomyopathy questionnaire^*^	63 ± 20	39 ± 22
Coexisting conditions-No. (%)		
Atrial fibrillation	16 (89)	24 (83)
Coronary artery disease	10 (56)	16 (55)
Prior CABG	7 (22)	9 (31)
Previous valvular intervention	9 (50)	14 (48)
Stroke/TIA	2 (11)	11 (38)
Chronic lung disease	5 (28)	7 (24)
Liver disease	1 (6)	9 (31)
Pacemaker/defibrillator	3 (17)	7 (24)

*Values are mean ± SD or n (%)*.

* *EFS data is for patients with paired data. CABG, coronary artery bypass grafting; TIA, transient ischemic attack*.

### Procedural outcomes

Of 18 compassionate care patients reported there were 2 failed procedures, due to RV perforation in 1 patient and 1 early device dislocation in another. At 30 days and 1 year follow up there were no mortalities reported (14).

The adjudicated data on 29 patients in the EFS trial reported 2 procedural failures, both related to RV perforations. Two devices were explanted, because of detachment of the anchor in one patient (surgical explant) and because of endocarditis in the other (percutaneous explant on day 21). There were two procedure related deaths, as a result of RV perforation and as a consequence of the surgical explant (15). Thirty day procedural outcomes are shown in Table 2.

**Table 2:** Thirty day outcomes.

**Outcome**	**Compassionate care (*n* = 18)**	**EFS trial (*n* = 29)**
Death	0	2 (7)
Stroke	0	0
Myocardial infarction	0	0
Device related cardiac surgery	1 (6)	3 (10)
Bleeding		
Life threatening	1 (6)	2 (7)
Major	1 (6)	4 (14)
Vascular complications, major	0	1 (3)
Acute kidney injury ≥stage 2	0	3 (10)
Pulmonary embolism	0	0
New pacemaker	0	0
Device associated infection	0	1 (3)

*Values are n (%)*.

### Echocardiographic results

In the EFS trial 25 patients were available for echocardiographic follow up at 30 days. Core lab evaluation of TR showed reductions of TR, that were mostly in the range of torrential TR at baseline, by 49% when evaluated by 2D/3D quantitative effective regurgitation orifice area (EROA) 2.1 ± 1.8 to 1.1 ± 0.9 cm^2^; and by 46% when using proximal isovelocity surface area (PISA) EROA 1.1 ± 0.6 to 0.6 ± 0.4 cm^2^. Compassionate care patients treated with the FORMA system experienced similar reductions in TR severity (reduction of vena contracta width from 12.1 ± 3.3 to 7.1 ± 2.2 mm at 30 days), these measurements were site reported. Despite the short follow up, reductions in RV sizes were seen in both cohorts. In the EFS trial, Annular and RV base diameters were reduced (4.4 ± 0.7 to 4.5 ± 0.9 cm, *P* = 0.57 and 5.9 ± 0.9 to 5.5 ± 1.0 cm, *P* = 0.02, respectively) by 30 days. In the compassionate care cohort similar reductions were observed (annular diameters: 4.6 ± 0.5 to 4.3 ± 0.5 cm, *P* = 0.04 and RV diameters: 5.4 ± 0.5 to 5.0 ± 0.5 cm, *P* = 0.02). The echocardiographic results are presented in Table 3.

**Table 3:** Echocardiographic results.

**Characteristic**	**Compassionate care (*n* = 18) baseline**	**30 Days**	***P*-value**	EFS trial (*n* = 25)^*^ baseline	**30 days**	***P*-value**
TR vena contracta, cm	1.2 ± 0.3	0.7 ± 0.2	<0.001	1.6 ± 0.5	1.1 ± 0.4	<0.001
Effective regurgitation orifice area, cm^2^	1.0 ± 0.6	0.4 ± 0.3	0.001	1.1 ± 0.6	0.6 ± 0.4	0.001
Tricuspid annular diameter, cm	4.6 ± 0.5	4.3 ± 0.5	0.09	4.4 ± 0.7	4.5 ± 0.9	0.58
RV diameter, base, cm	5.4 ± 0.5	5.0 ± 0.5	0.06	5.9 ± 0.9	5.5 ± 1.0	0.02
TAPSE, cm	1.5 ± 0.5	1.4 ± 0.3	0.44	1.4 ± 0.4	1.5 ± 0.4	0.59
Left ventricular ejection fraction, %	59 ± 9	61 ± 9	0.52	55.9 ± 13.8	58.6 ± 12.9	0.07

*Values are mean ± SD or n (%)*.

* *EFS data is for patients with paired data. TAPSE, tricuspid annular plane systolic excursion*.

### Functional outcomes

**Figure 3:**
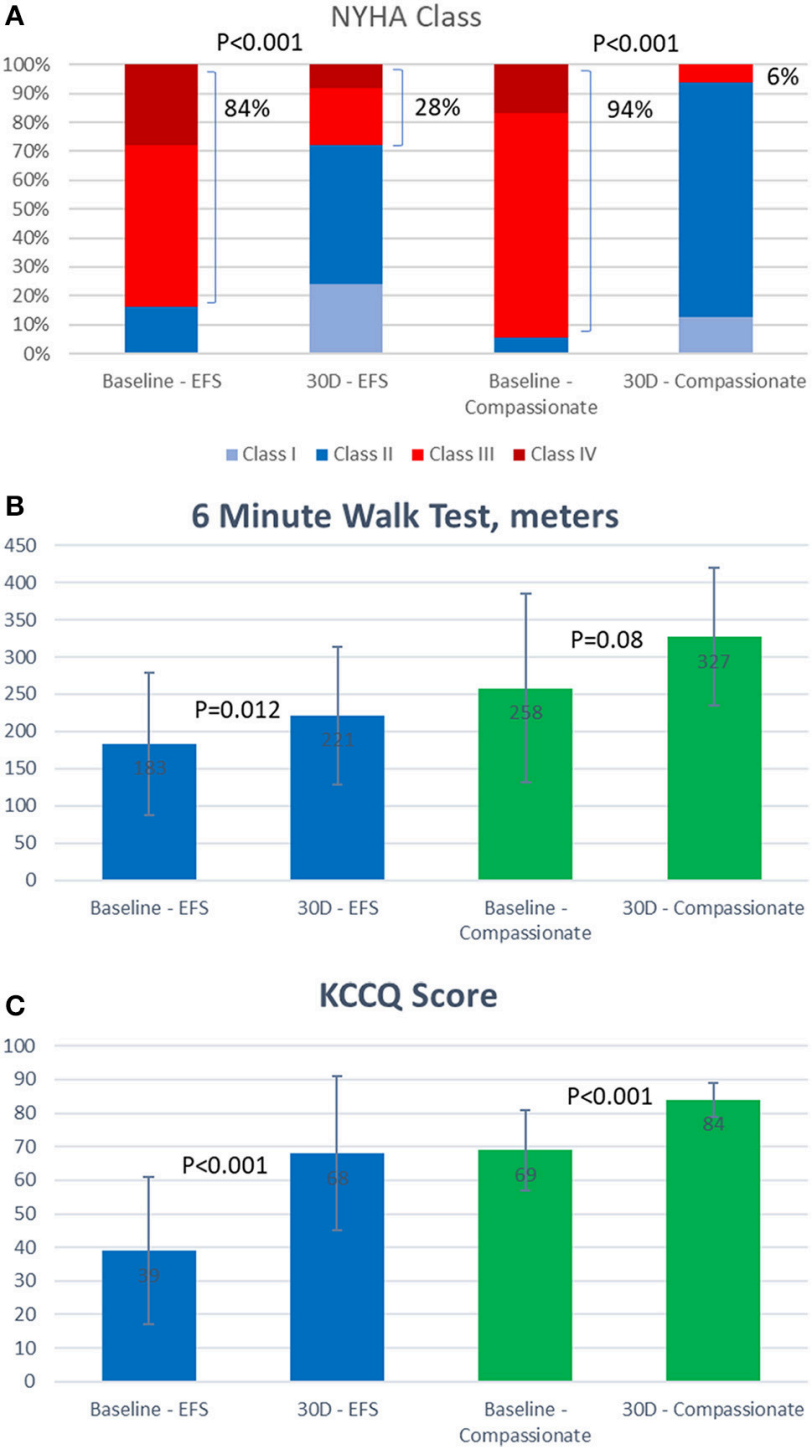
Thirty day functional outcomes of patients treated with the FORMA device. **(A)** New York Heart Association (NYHA) class. **(B)** Six minute walk test results (meter). **(C)** Kansas City Cardiomyopathy Questionnaire (KCCQ) score.

Patients treated successfully with the FORMA experienced significant clinical improvements by 30 days in both the EFS trial and the compassionate cohort (Figure 3). In the compassionate cohort these improvements were sustained in the patients with 1 year follow up (*n* = 15).

In both cohorts the vast majority of patients (84 and 94%) were in New York heart association (NYHA) class III or IV at baseline, this was reduced by 30 days to 28% in the EFS trial patients and 6% in the compassionate cohort. Seventy-nine percent of the compassionate care patients remained in NYHA class I or II at 1 year. At 30 days, the average 6 min walk test improved by 39 meters in the EFS trial and 69 meters in the compassionate cohort. Similarly, the average Kansas City cardiomyopathy questionnaire heart failure score assessed at 30 days, improved by 29 points in the EFS trial and by 15 points in the compassionate cohort.

## Conclusions

The tricuspid valve, long considered the “forgotten valve,” is becoming a recognized target for transcatheter therapies aimed at treating symptomatic patients for whom there are no other viable options.

A striking similarity between the various devices is their positive clinical effect on the patients. Despite only modest reductions in the severity of TR reported in most studies, almost all reports of transcatheter devices show significant improvements in clinical outcomes such as NYHA class, 6 min walk test and heart failure scores. This is especially impressive given the fact that the patients treated generally extremely sick, with multiple co-morbidities.

The FORMA device acts as a spacer and thus seems independent of the size and shape of the annulus and the valve leaflets. The clinical experience with this device has been achieved in patients with extreme EROA's. Nickenig et al reported a PISA EROA of 0.9 ± 0.4 cm^2^ in patients treated with the Mitraclip device (9) and Hahn et al. reported a PISA EROA of 0.51 ± 0.16 cm^2^ in patients treated with the Trialign device (10). In the FORMA EFS trial, the mean PISA EROA was 1.1 ± 0.6 cm^2^, with some patients having EROA's even larger than 2.0 cm^2^.

Data on specific characteristics that may define a patient population more likely to respond to the FORMA device is still not available. Access to the left subclavian vein is required, patients with occluded veins may not be suitable for this device. Additionally, patients with pacemaker leads that are adherent to the valve leaflet were excluded from the EFS trial, as this was considered to prevent coaptation of the leaflets with the spacer.

Anchoring the device to the RV is an important step of the procedure. The anchoring process requires adequate imaging and careful manipulation of the delivery system to avoid injuring the thin-walled RV. Refinements in implantation techniques and devices are needed to ensure the success of this step in a broader population of patients outside of controlled trials.

Further evaluation of the FORMA device is ongoing with expected inclusion of additional patients and refinements in anchoring techniques.

## Author contributions

All authors listed have made a substantial, direct and intellectual contribution to the work, and approved it for publication.

### Conflict of interest statement

GP and DD are both consultants of Edwards Lifesciences.
